# Subtropical grass pollen allergens are important for allergic respiratory diseases in subtropical regions

**DOI:** 10.1186/2045-7022-2-4

**Published:** 2012-03-05

**Authors:** Janet Mary Davies, Hongzhuo Li, Melissa Green, Michelle Towers, John Warrick Upham

**Affiliations:** 1 Lung and Allergy Research Centre, School of Medicine, The University of Queensland, Princess Alexandra Hospital Clinical Division, Woolloongabba, QLD 4076, Australia; 2 Department of Respiratory Medicine, Princess Alexandra Hospital, Woolloongabba, QLD 4102, Australia

**Keywords:** Grass pollen, Allergic rhinitis, IgE, Bahia grass pollen, Bermuda grass pollen, Ryegrass pollen

## Abstract

**Background:**

Grass pollen allergens are a major cause of allergic respiratory disease but traditionally prescribing practice for grass pollen allergen-specific immunotherapy has favoured pollen extracts of temperate grasses. Here we aim to compare allergy to subtropical and temperate grass pollens in patients with allergic rhinitis from a subtropical region of Australia.

**Methods:**

Sensitization to pollen extracts of the subtropical Bahia grass (*Paspalum notatum*), Johnson grass (*Sorghum halepense*) and Bermuda grass (*Cynodon dactylon*) as well as the temperate Ryegrass (*Lolium perenne*) were measured by skin prick in 233 subjects from Brisbane. Grass pollen-specific IgE reactivity was tested by ELISA and cross-inhibition ELISA.

**Results:**

Patients with grass pollen allergy from a subtropical region showed higher skin prick diameters with subtropical Bahia grass and Bermuda grass pollens than with Johnson grass and Ryegrass pollens. IgE reactivity was higher with pollen of Bahia grass than Bermuda grass, Johnson grass and Ryegrass. Patients showed asymmetric cross-inhibition of IgE reactivity with subtropical grass pollens that was not blocked by temperate grass pollen allergens indicating the presence of species-specific IgE binding sites of subtropical grass pollen allergens that are not represented in temperate grass pollens.

**Conclusions:**

Subtropical grass pollens are more important allergen sources than temperate grass pollens for patients from a subtropical region. Targeting allergen-specific immunotherapy to subtropical grass pollen allergens in patients with allergic rhinitis in subtropical regions could improve treatment efficacy thereby reducing the burden of allergic rhinitis and asthma.

## Background

Grass pollens are amongst the most frequently recognised aeroallergens worldwide [1,2]. Allergic rhinitis is an important health problem causing itching, fatigue, decrease in quality of life, reduced productivity and complications such as sleep apnoea and sinusitis [3]. Allergic rhinitis poses a significant ongoing health burden per se but it carries additional adverse consequences such as increasing the risk of developing asthma and being associated with poor asthma control [4]. Recent studies have established an association between hospital admissions for asthma and airborne grass pollen allergen levels [5] as well as a causal relationship between grass pollen challenge and asthma exacerbation [6]. Furthermore, epidemics of thunderstorm-induced asthma due to grass pollen allergy have been well documented in Australia and elsewhere [7-11]. The most recent epidemic that occurred in November 2010 in Melbourne Australia, followed days with extremely high grass pollen counts and affected people without a previous history of asthma [7].

**Figure 1:**
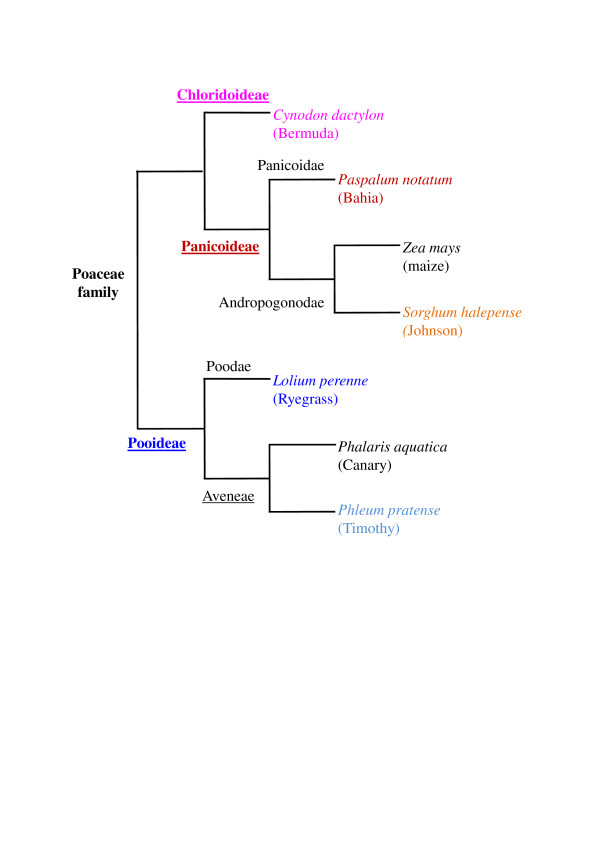
**Phylogenetic tree of Poaceae grasses with allergenic pollens**. Subfamily levels are underlined and clinically relevant species whose pollen investigated herein are coloured.

Despite the clinical importance of grass pollen allergy in allergic respiratory diseases, it has not been established which of the grass pollens are most relevant to Australian environments, with the exception of Ryegrass (*Lolium perenne*) which is the major clinically relevant allergenic grass pollen in temperate regions of Australia [12,13]. The subtropical climate throughout parts of Queensland supports a wider range of grasses than some other regions with predominance of many species of subtropical grasses from several botanical subfamilies of Poaceae grasses (Figure 1). Environmental surveys on grass distribution in Queensland in the 1960s indicated that Bahia grass (*Paspalum notatum*), Johnson grass (*Sorghum halepense*) and Bermuda grass (*Cynodon dactylon*) were among the most abundantly observed grasses [14], although Ryegrass is also observed in south east Queensland[15]. Aerobiological pollen sampling conducted in Brisbane in 1990s showed that grass pollens contribute the major part (65%) of airborne pollen load [16]. The environmental distribution of subtropical grasses is likely to spread with climatic increases in temperature and carbon dioxide levels, escalating the importance of their contribution to allergic respiratory diseases in future [17]. However, there is very little clinical data on allergic sensitization to grass pollens in patients with allergic rhinitis in subtropical regions. Such information is likely to be valuable for optimal use of allergen specific immunotherapy in Australia as well as other warmer regions of the world, including the southern states of America where these types of subtropical grasses are also important allergen sources [18,19].

Here we examine allergic sensitization by skin prick testing and assess IgE reactivity in subjects from Brisbane to a panel of subtropical grass pollens from separate botanical subfamilies; Bahia, Johnson and Bermuda grasses, in comparison with pollen of the temperate Ryegrass. The relative strength of IgE reactivity with the different grass pollen allergens and presence of species-specific IgE reactivity with subtropical grass pollen allergens are examined.

## Methods

### Study participants

**Figure 2:**
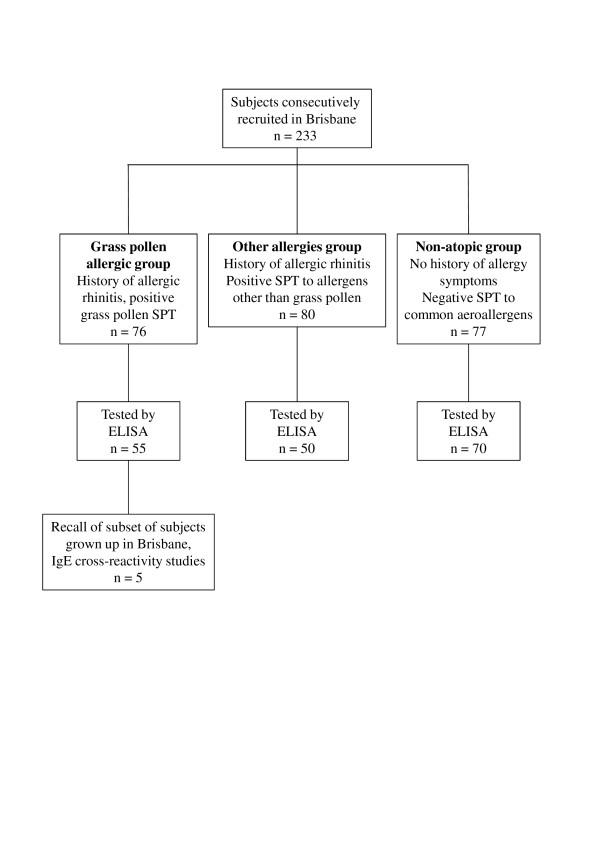
**Inclusion of participants to subject groups**. The number recruited to the grass pollen allergic group, other allergies group, or the non-atopic group based on clinical history and skin prick test (SPT) are given. The number in each group from whom plasma was available for IgE testing is given in the next level. A subset of grass pollen-allergic subjects were recalled for IgE cross-reactivity studies.

Two hundred and thirty three participants were recruited consecutively from amongst laboratory and healthcare workers or patients who were living in the Brisbane region presenting to an Immunology or Respiratory clinic at the Princess Alexandra Hospital. Written informed consent was obtained from each participant; the study was approved by the Metro South Human Research Ethics Committee. Symptoms of allergies and asthma were recorded and participants were assessed for allergic sensitivity by skin prick testing and ELISA with a panel of common aeroallergens including pollen extracts of Bahia grass, Johnson grass, Bermuda grass or Ryegrass. Subjects were assigned to the grass pollen allergic group based on clinical history of allergic rhinitis and SPT to any grass pollen extract, the other allergy group based on clinical history of allergy and SPT response to allergens other than grass pollen, or the non-atopic group for those without clinical history of allergy and negative SPT (Figure 2). Plasma and sera were obtained from study participants by venepuncture.

### Skin prick tests

Skin prick testing were performed according to the guidelines of the Australian Society for Clinical Immunology and Allergy [20]. Briefly, droplets of commercially supplied allergen extracts (Holister Steir, Spokane, WA, USA) that were registered by the Therapeutic Goods Authority and widely used in standard clinical practice in Australia, were placed on inside forearm and the skin was superficially pricked with a lancet (ALK-Abello, Horsholm, Denmark). Reactions were measured at 15 min post prick. Patients were requested to withdraw anti-histamine medication for four days prior to testing. Histamine and saline solutions were used as positive and negative controls respectively. Wheals equal or greater than 3 mm in average diameter were considered positive. The allergen extracts included Bermuda (standardized at 10,000 BAU/ml), Ryegrass (standardized at 10,000 BAU/ml), Bahia (1/10 weight/volume), Johnson (1/10 weight/volume) and Southern grass mix (Kentucky Bluegrass *Poa pratensis*, Orchard *Dactylis glomerata*, Redtop *Agrostis gigantea*, Timothy *Phleum pratense*, Sweet Vernal *Anthoxanthum odoratum*, Bermuda, Johnson at 1/10 volume/volume) grass pollen extracts, house dust mites (*Dermataphagoides pterynisius*, standardized at 30,000 AU/ml), cat hair (standardized at 10,000 AU/ml), moulds (*Apergillus fumigates *1/10 weight/volume, and *Alternaria tenuis *111.33 Units/ml of Ant. E.), and Ragweed pollen (Giant, Short and Western mixture, 1/20 weight/volume).

### Allergen extracts

Proteins from non-defatted pollen grains of Ryegrass, Bahia, Timothy, Johnson and Bermuda grasses (Greer Laboratories, Lenoir NC, USA) were extracted in phosphate buffered saline with complete protease inhibitor cocktail (Roche Diagnostics, Basel, Switzerland) for three hours on a rotating wheel at 4°C and clarified as previously described [21]. Raw peanut (*Arachis hypogaea *extract) was prepared as a control allergen [22].

### ELISA

Plasma samples diluted 1/10 were tested by ELISA with microtiter plate wells coated with 5 μg/ml of whole grass pollen extract as described previously [23]. Inhibition ELISA assays were conducted for individual sera diluted to a concentration within the linear phase of titration curves of IgE reactivity with grass pollen extracts giving approximately 1 Optical Density units (OD). Reciprocal cross-inhibition ELISA were performed to test the capacity of various pollen extracts or crude peanut extract to block serum IgE reactivity with each of the four grass pollen extracts from Ryegrass, Bahia, Johnson and Bermuda grasses as previously described [23]. Briefly, inhibitor allergen extracts including Ryegrass, Bahia, Johnson, Bermuda and Timothy grass pollens, or peanut extract control, (0.05 to 50 μg/mL) were pre-incubated with diluted sera for 90 min prior to addition in triplicate to antigen-coated and blocked microtiter plate wells.

### Statistics

Data was assessed for normality by Kolmogorov-Smirnov test and assessed for differences by Friedman's ANOVA with Dunn's multiple comparison tests. P values less than 0.05 (*), < 0.01 (**), or < 0.001 (***) were considered significant. Correlations of SPT and IgE reactivity between different grass pollens were determined by Spearman's rank test for paired data.

## Results

### Allergic sensitivity to different grass pollens assessed by skin prick test

Participants were recruited from amongst patients presenting to an Immunology or Respiratory clinic at a major public hospital in Brisbane, as well as amongst healthcare and laboratory workers. Seventy seven participants were skin prick test (SPT) negative to all aeroallergens tested. Eighty subjects were SPT positive to allergens other than grass pollens with house dust mite being the most frequently recognized allergen. Seventy six of the 233 participants assessed by skin prick tests showed positive reactions to at least one grass pollen extract (Ryegrass, Bahia, Johnson, Bermuda or Southern grass mix). There were 48 grass pollen-allergic subjects for whom SPT data was available for each of Ryegrass, Bahia, Johnson and Bermuda grass pollens. Of these 48 subjects there were eight (16%) who only showed positive SPT to subtropical grasses, whereas three (6%) showed a positive SPT to Ryegrass pollen only.

**Figure 3:**
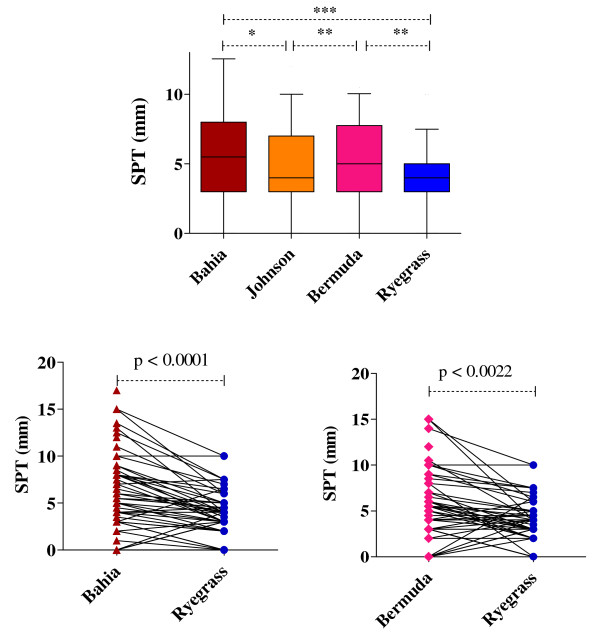
**Allergic sensitization to different grass pollen extracts**. A. Average skin prick test diameters for subtropical Bahia, Johnson and Bermuda grass pollens and temperate Ryegrass pollen. Box and whisker plots show median, interquartile range, 10^th ^and 90^th ^percentiles and minimal and maximal individual values for data from 48 grass pollen allergic donors from Brisbane. Significant differences by Friedman ANOVA and Dunn's multiple comparison test. B and C. Pairwise data for individual patient sensitivity comparing either Bahia or Bermuda grass pollen with Ryegrass pollen.

SPT wheals were significantly larger with Bahia and Bermuda grass pollens than with Ryegrass pollen (Figure 3). Interestingly, although Johnson grass is also a subtropical grass of the Panicoideae subfamily, SPT wheals in response to Johnson grass pollen were smaller than with Bahia grass pollen (Figure 3).

There were significant correlations in allergic sensitivity between each of the grass pollens (Table 1). The strongest correlation was between Bahia and Johnson grass pollens (r = 0.841, 95% confidence intervals (CI) of 0.729-0.909) whereas the weakest correlation was between Ryegrass and Bermuda grass pollens (r = 0.516, CI 0.263-0.702).

**Table 1:** Correlation between skin prick reactivity with grass pollens in subjects with grass pollen allergy in Brisbane

	Johnson	Bahia	Bermuda	Ryegrass
Johnson	1.0			
Bahia	0.841 (0.729-0.909)	1.0		
Bermuda	0.825 (0.704-0.900)	0.779 (0.639-0.870)	1.0	
Ryegrass	0.687 (0.494-0.816)	0.717 (0.538-0.835)	0.516 (0.263-0.702)	1.0

Spearman's r with 95% confidence intervals shown in brackets. All correlations were significant (*p *< 0.0001) based on Gaussian approximations.

### Plasma IgE reactivity with different grass pollen extracts

IgE reactivity with each of the grass pollens was tested by ELISA in 175 subjects for whom plasma was available. The IgE reactivity of the 70 SPT-negative, non-atopic donors was used to define the normal range. Concentrations greater than three standard deviations above the mean IgE reactivity of normal subjects were considered positive; these cut off values were as follows - Bahia grass (0.190 OD; Johnson grass (0.163 OD), Bermuda grass (0.129 OD) and Ryegrass (0.187 OD). Using this definition, of 50 subjects with allergies other than grass pollen, there were one, two, two and four subjects who tested positive for IgE reactivity with Bermuda, Johnson, Bahia and Ryegrass pollen, respectively. Forty five of 55 (81%) of grass pollen-allergic subjects with positive SPT to any grass pollen showed IgE reactivity with at least one of the four grass pollens by ELISA. There were ten subjects (18%) who only showed IgE reactivity with subtropical grass pollens whereas only four (7%) subjects showed IgE reactivity with Ryegrass pollen only. Mostly, subjects showed IgE reactivity with more than one grass pollen but there were differences in the levels of IgE reactivity.

**Figure 4:**
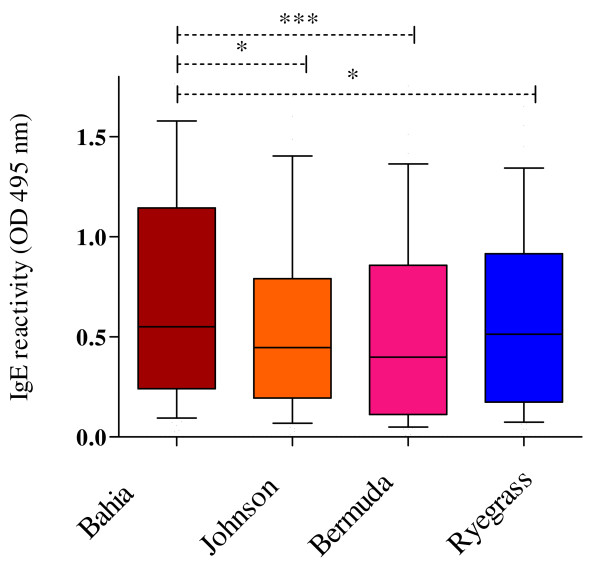
**Plasma IgE reactivity with pollen of subtropical Bahia, Johnson and Bermuda grasses and temperate Ryegrass pollen**. Box and whisker plots show median, interquartile range, 10^th ^and 90^th ^percentiles and minimal and maximal individual values for data from 55 grass pollen allergic donors from Brisbane. Significant differences by Friedman ANOVA and Dunn's multiple comparison test. Data on IgE reactivity with Bahia and Ryegrass pollen for 47 of these subjects were published in ref 21.

The IgE reactivity of the grass pollen-allergic donors was higher with Bahia grass pollens than with Johnson and Ryegrass pollens (Figure 4). IgE reactivity with Bahia grass pollen was significantly higher than with Bermuda grass pollen (Figure 4).

Plasma IgE reactivity with each of the grass pollens were well correlated amongst the grass pollens (Table 2). However, the correlation between Bahia and Johnson grass pollens (r = 0.884, CI 0.806-0.932) was higher than between Ryegrass and Bermuda grass pollens (r = 0.641, CI 0.446-0.778).

**Table 2:** Correlation between plasma IgE reactivity with grass pollens in subjects with grass pollen allergy in Brisbane

	Johnson	Bahia	Bermuda	Ryegrass
Johnson	1.0			
Bahia	0.884 (0.806-0.932)	1.0		
Bermuda	0.854 (0.758-0.914)	0.805 (0.682-0.884)	1.0-	
Ryegrass	0.815 (0.697-0.890)	0.766 (0.624-0.859)	0.641 (0.446-0.778)	1.0

Spearman's r with 95% confidence intervals shown in brackets. All correlations were significant (*p *< 0.0001) based on Gaussian approximations.

### Species-specific serum IgE reactivity with subtropical grass pollens

**Figure 5:**
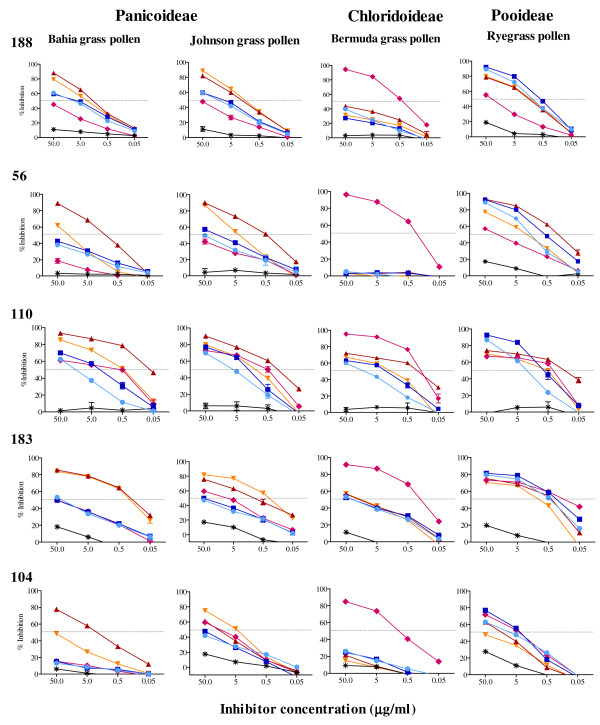
**Cross-inhibition ELISA of serum IgE reactivity with different grass pollen extracts**. Serum numbers are given on the top right of each row and the target grass pollen extract with botanical subfamilies are given for each column of graphs. Inhibitors were added to diluted sera from 0.05 to 50 μg/ml (red triangle symbol, Bahia grass; orange inverted triangle symbol, Johnson grass, pink diamond symbol, Bermuda grass, blue square symbol, Ryegrass, light blue circle symbol, Timothy grass and * Peanut extract; a non-grass pollen, allergen control). Data is expressed as the percentage inhibition of uninhibited IgE reactivity with standard errors for three technical replicates shown. The relative avidity of interaction between serum IgE and a target grass pollen is indicated by the maximum level of inhibition achieved with 10 fold excess of pollen inhibitor in solution over target solid-phase pollen allergen, as well as the concentration of pollen inhibitor required to block 50% (IC50; indicated dotted line) of IgE reactivity. Pooled data from four of these donors for inhibition of IgE reactivity with Bahia and Ryegrass pollen was published in reference 21.

Reciprocal cross-inhibition assays were performed in a subset of subjects known to have grown up in the Brisbane area and who would represent patients primarily exposed to subtropical grass pollens, in order to compare the strength of IgE reactivity with subtropical versus temperate grass pollen allergens (Figure 5). The relative avidity of interaction between serum IgE and a particular grass pollen is indicated by the maximum level of inhibition achieved with 10 fold excess of pollen inhibitor in solution over solid-phase pollen allergen, as well as the concentration of pollen inhibitor required to block 50% (IC50) of IgE reactivity. Mostly, IgE reactivity with Bermuda grass pollen was only blocked effectively by Bermuda grass pollen whilst pollen of the Bahia and Ryegrass pollens, failed to reach 50% inhibition indicating the presence of unique IgE binding sites on Bermuda grass pollen allergens. In contrast, IgE reactivity with Ryegrass pollen, in most subjects, was inhibited by other grass pollens to a comparable level as Ryegrass pollen, indicating a lack of specific IgE to unique epitopes of Ryegrass pollen allergen. For sera 188 and 56, Bermuda grass pollen had lower capacity to inhibit IgE reactivity with Ryegrass pollen than the other grass pollens indicating low avidity IgE binding to epitopes of Ryegrass pollen allergens or difference in allergen composition between Ryegrass and Bermuda grass pollen. In all the subjects, IgE reactivity with Bahia and Johnson grass pollens showed higher maximal inhibition by Bahia and Johnson grass pollens than by the other grass pollens, indicating species-specific IgE reactivity with these subtropical grass pollens. There was partial inhibition of IgE reactivity with Bahia grass pollen by Ryegrass and Timothy grass pollen in most subjects (except 104), but with lower maximal inhibition and 100 to 1000 fold higher concentrations of inhibitor required to yield 50% inhibition (IC50), indicating less avid IgE binding to temperate grass pollens. Notably, the two pairs of pollens from the one botanical subfamily mostly showed similar cross-inhibition capacity as each other with almost identical inhibition curves for Ryegrass and Timothy grass pollens. However, in sera of three subjects, 56, 110 and 104, Johnson grass pollen did not inhibit IgE reactivity with the other grass pollens to the same extent as Bahia grass pollen.

## Discussion

Hitherto grass pollen allergy has been primarily studied in subjects living in regions with temperate climates such as parts of Europe and North America where Timothy grass pollen is the predominant clinically important grass pollen. Ryegrass pollen has been considered to be the most clinically important grass pollen allergen in Australia [12,13]. However, subtropical grass pollens also make a contribution as significant sources of grass pollen allergen in Australia [21,24,25]. This is the first study to undertake a detailed comparison of allergic sensitization to grass pollen allergens in patients from a subtropical region. A strength of the study is that it examined pollens sourced from both Panicoideae and Chloridoideae subfamilies of subtropical grasses. The outcomes of this investigation provide evidence that patients recruited from a subtropical region of Brisbane show significantly higher skin prick test and plasma IgE reactivity with subtropical grass pollens than Ryegrass pollen and the presence of unique epitopes in subtropical grass pollens recognised by serum IgE.

Here we show marked differences in the relative avidity of IgE binding between different grass pollens in dose responsive four-way reciprocal cross-inhibition experiments. Other studies of patients from temperate regions focused on inhibition of IgE reactivity with Timothy grass pollen extract or its allergenic components with an excess of pollen inhibitor and reported maximal inhibition capacity but not relative avidity as revealed by decreasing doses of pollen inhibitor [26,27]. Pooled sera from volunteers in the armed forces in the US, of unknown origin, revealed asymmetric cross-reactivity and unique allergenicity of Bahia and Bermuda grass pollen [27]. In keeping with this, our data from individual patients from a subtropical region showed higher avidity of serum IgE reactivity with Bahia, Johnson, Bermuda grass pollens than with Ryegrass pollen. The inability of temperate Ryegrass and Timothy grass pollens, to fully inhibit IgE reactivity with pollen of subtropical species, Bahia, Johnson and Bermuda grasses, confirms the presence of unique epitopes in subtropical grass pollen allergens that are not represented in pollen allergens of the temperate grasses.

There were differences in the degree of correlation of SPT sensitivity (Table 1) and plasma IgE reactivity (Table 2) between Ryegrass and Bermuda grass pollen. This is consistent with European studies showing that specific IgE levels to Bermuda, Bahia and Johnson grass pollens were not tightly correlated with IgE reactivity to Timothy grass pollen as other temperate grass pollens [28]. Data from another large panel of European subjects showed markedly lower correlation of IgE reactivity between Timothy and Bermuda grass pollens than amongst the temperate grass species including Timothy and Ryegrass [29]. This current study extends our earlier work showing a trend for higher IgE reactivity with Bahia grass pollen than Ryegrass pollen in 47 grass pollen-allergic subjects from Brisbane [23]. Here, with comparative data IgE reactivity with four grass pollens for 55 grass pollen allergic donors the observation of higher IgE reactivity with Bahia grass pollen achieved statistical significance.

There are known to be differences in allergen composition amongst grass pollens. Pollens of different species of temperate grasses all contain several major allergen families including group 1, 2, 4 and 5 whereas the subtropical grass pollens lack the group 2 and 5 allergen families [30]. There are substantial variations in the two major Pooideae pollen allergens of group 1 and group 5, even amongst five phylogenetically related temperate grass species [26,31-34]. The variability between allergens of more distantly related grass pollens of the Chloridoideae and Panicoideae subfamilies, and allergens of the Pooideae temperate grass subfamily are consequently likely to be substantially greater. For example similarity amongst group 1 allergens of temperate grass pollens is higher than between subtropical and temperate grass pollen allergens; Lol p 1 of Ryegrass and Phl p 1 of Timothy grass share from 86-88% amino acid identity, depending on the isoforms of the allergens, whereas the major group 1 allergen of Bahia grass pollen, Pas n 1, has only 66 and 64% amino acid identity with Lol p 1 or Phl p 1, respectively [33,35]. It is not entirely unexpected then, that grass pollen-allergic subjects from a subtropical region exhibit species-specific IgE epitope recognition of subtropical grass pollens.

The difference in allergic sensitivity and allergen recognition depends not only on biological differences between the pollens but also upon the origin of the patients investigated. Our observations with patients from a subtropical region is in contrast to IgE reactivity of patients from the temperate region city of Melbourne, who showed substantially higher IgE reactivity with pollens of Ryegrass compared with Bahia and Bermuda grasses [23]. Similarly, the asymmetric cross-inhibition data of Brisbane patients showed the opposite trend to inhibition data from Melbourne subjects where IgE reactivity with pollen of Bahia was fully blocked by Ryegrass grass pollen but not vice versa [23]. Regional variation in serological recognition of pollen allergens could be attributable to differences in exposure to particular grasses in diverse climatic regions, however data on the environmental distribution of pollens from particular grasses is lacking because of the inability to distinguish different types of grass pollens in aerobiological samples by current microscopy-based techniques. Although the numbers of cross-inhibition experiments are small, the data presented here nonetheless demonstrates that individuals produce IgE that recognise unique antigenic determinants within particular grass pollen allergens and this recognition appears to vary regionally. Regional variation in IgE reactivity with grass pollen allergens has been also been described in Europe [36]and North America [18].

We also sought to compare the allergic sensitivity and IgE reactivity amongst subtropical grass pollens. The same heterogeneity evident within temperate grass pollen allergens discussed earlier might be expected for pollen allergens of the subfamilies of subtropical grasses. Indeed we observed Johnson grass pollen elicited lower SPT and plasma IgE reactivity than Bahia grass pollen as well as lower capacity to inhibit serum IgE reactivity with other grass pollens, including Bahia grass pollen suggesting that there may be allergenic differences between these Panicoideae grass pollens. It is worth considering that although both these grasses belong to the one subfamily they align to different subtribes, Paniceae for Bahia grass and Andropogoneae for Johnson grass, which may explain why these grass pollens were not interchangeable. A difference between Bahia and Bermuda grass pollens was not evident by SPT but Bahia grass pollen showed higher levels of IgE reactivity than Bermuda grass pollen. We noted that Bermuda grass pollen appeared to be more allergenically distinct from Ryegrass than Bahia grass pollen since in three of five donors IgE reactivity with Bermuda was only substantially inhibited by itself and it had only limited capacity to block IgE reactivity with Ryegrass, Johnson or Bahia in some subjects. Also, SPT and plasma IgE reactivity with Bermuda showed the least correlation with Ryegrass suggesting greater allergenic differences between Bermuda and Ryegrass pollens than between Bahia and Ryegrass. However, further studies are warranted to confirm this observation with a wider representation of patients from subtropical regions.

The outcomes of this study are likely to impact upon the clinical efficacy of grass pollen allergen-specific immunotherapy in patients from a subtropical region. Whilst for many treatment for allergic rhinitis can be managed by allergen avoidance and pharmacotherapy, for others with more severe disease, specific immunotherapy is indicated [37]. Grass pollen allergen immunotherapy for prevention of hay fever has been in clinical use for 100 years [38], and provides relief from symptoms, limits the acquisition of new allergic sensitivities, decreases cost of medication in the long term and may reduce the risk of developing asthma in older children [39,40]. However, many immunotherapy reagents, in particular the new generation of allergy vaccine tablets for sublingual immunotherapy, are based solely on temperate grass pollen allergens [41,42]. Given the species-specific pollen IgE reactivity observed here, it is likely that tailoring of grass pollen immunotherapy to subtropical grass pollen allergen vaccines would improve treatment efficacy in patients primarily sensitized to subtropical grass pollens such as Bahia and Bermuda grasses.

## Conclusions

Here we demonstrate higher SPT and IgE reactivity with subtropical grass pollens than Ryegrass in patients from a subtropical region. We also observed incomplete and asymmetric cross-inhibition of serum IgE between subtropical grass pollens and the Ryegrass pollen allergen in patients with grass pollen allergy in Brisbane. Further studies are warranted to assess difference in allergic sensitization in other parts of Australia, including arid and wet tropical regions where there are hotter climates and subtropical grass pollens are likely to contribute to grass pollen sensitization. It will be necessary in the longer term to investigate whether differences observed here in IgE recognition of subtropical and temperate grass pollen affect the efficacy of immunotherapy for patients primarily sensitized to subtropical grass pollen allergens in Australia and other regions with similar climates

## Abbreviations

ELISA: Enzyme-linked immunosorbant assay; IC50: Inhibitor concentration giving 50% inhibition; IgE: Immunoglobulin E; OD: Optical density units; SPT: Skin prick test.

## Competing interests

JMD has received grants from the Asthma Foundation of Australia, Australian Society for Clinical Immunology and Allergy, The University of Queensland and the National Health and Medical Research Council of Australia, has collaborations with Sullivan Nicolaides Pathology (Queensland) and Phadia Pty Ltd (Uppsala, Sweden), and is an inventor on an Australian and US patent on "Novel immunogenic molecules and uses thereof (Bahia grass)". JMD has also received consulting fees from Stallergenes Pty Ltd (Antony, France) and has a collaborative research agreement with this company. MT has received Honoraria from Novartis for asthma nurse education presentations. JWU has been paid Honoraria from Astra Zeneca to present updates on asthma at approved medical education events.

## Authors' contributions

JD was the senior author of this manuscript. JD designed the study, analysed data, prepared and edited the manuscript. HZL performed the ELISA experiments. MG and MT assisted with patient recruitment and skin prick testing. JWU was involved with subject recruitment and provided intellectual input to the study. All authors read and approved the final manuscript.
